# Supplementary material for: SARS-CoV-2 Omicron Variant Neutralization after Third Dose Vaccination in PLWH
Source: Viruses. 2022 Aug 3;14(8):1710. doi: 10.3390/v14081710 (PMC9415025; doi:10.3390/v14081710)
Supplement: Supplementary file 1 [file viruses-14-01710-s001.zip › viruses-1787290-SI.pdf]

SARS-CoV-2 Omicron Variant Neutralization after third dose Vaccination in PLWH

Alessandra Vergori[1*], Alessandro Cozzi-Lepri[2], Giulia Matusali[3], Francesca Colavita[3], Stefania Cicalini[1], Paola Gallì[4], Anna Rosa Garbuglia[3], Marisa Fusto[1], Vincenzo Puro[5], Enrico Girardi[6], Francesco Vaia[7], Andrea Antinori[1] on behalf of the HIV-VAC study group

[1]*HIV/AIDS Unit, National Institute for Infectious Diseases Lazzaro Spallanzani IRCCS, Rome, Italy*
[2]*Centre for Clinical Research, Epidemiology, Modelling and Evaluation (CREME), Institute for Global Health, UCL, London, UK*
[3]*Laboratory of Virology, National Institute for Infectious Diseases Lazzaro Spallanzani IRCCS, Rome, Italy*
[4]*Health Direction, National Institute for Infectious Diseases Lazzaro Spallanzani IRCCS, Rome, Italy*
[5]*Risk Management Unit, National Institute for Infectious Diseases Lazzaro Spallanzani IRCCS, Rome, Italy*
[6]*Scientific Direction, National Institute for Infectious Diseases Lazzaro Spallanzani IRCCS, Rome, Italy*
[7]*General Direction, National Institute for Infectious Diseases Lazzaro Spallanzani IRCCS, Rome, Italy*

Corresponding author

Alessandra Vergori, MD
e-mail: alessandra.vergori@inmi.it
Fax: +39 06 55170477
HIV/AIDS Unit
National Institute for Infectious Diseases Lazzaro Spallanzani IRCCS
Phone: +39 06 55170546
Via Portuense 292, 00149 Roma, Italy

Supplementary Table S1. Main descriptive characteristics of study population(n=134)

	HIV low (N=81)			HIV high (N=25)	HCWs (N=28)
	CD4 <200/mmc (N=27)	CD4 201-500/mmc (N=29)	CD4 >500/mmc (N=25)	CD4 >500/mmc	-
Age, median (IQR)	60 (52-68)	56 (47-61)	56 (48-60)	45 (42-52)	46.5 (34-53)
Gender F, n(%)	7 (8.6)	4 (4.9)	4 (4.9)	4 (2.9)	12 (8.9)
AIDS, n(%)	7 (25.9)	22 (75.8)	24 (96)	0 (0)	N/A
Time interval between second and third dose vaccine, days, median (IQR)	157 (154-160)	157 (154-159)	151 (150-154)	182(177-186)	283 (IQR 278–289)

Supplementary Table S2. Contrast of PLWH with high CD4 nadir vs HCWs from fitting a truncated regression in $\log_2$ scale (Panel A); Contrast of PLWH with high and low CD4 nadir (Panel B); Contrast by CD4 group in low CD4 nadir group (Panel C).

PANEL A	Unadjusted and adjusted mean difference at T1 in nABs comparing Omicron vs. D614G neutralizations (log2 scale)			
	Unadjusted Mean (95% CI)	p-value	Adjusted* mean (95% CI)	p-value
HCW	0		0	
HIV high CD4 nadir	0.36 (-0.41, 1.14)	0.358	0.40 (-1.64, 2.43)	0.703

*adjusted for value D614G at T0, age, gender and time difference between T-1 and T0

PANEL B	Unadjusted and adjusted mean difference at T1 in nABs comparing Omicron vs. D614G neutralizations (log2 scale)			
	Unadjusted Mean (95% CI)	p-value	Adjusted* mean (95% CI)	p-value
	0		0	
HIV low CD4 nadir				
HIV high CD4 nadir	-0.17 (-0.72, 0.39)	0.554	0.23 (-0.44, 0.91)	0.497

*adjusted for value D614G at T0, age, gender and time difference between T-1 and T0

PANEL C	Unadjusted and adjusted mean difference at T1 in nABs comparing Omicron vs. D614G neutralizations (log2 scale)			
	Unadjusted Mean (95% CI)	p-value	Adjusted* mean (95% CI)	p-value
501+	0		0	
201-500	-0.64 (-1.29, 0.01)	0.055	-0.80 (-1.52, -0.08)	0.029
0-200	0.07 (-0.66, 0.80)	0.857	-0.17 (-0.95, 0.62)	0.679

*adjusted for value D614G at T0, age, gender and time difference between T-1 and T0
